# Association Between Proton Pump Inhibitor Use During Early Pregnancy and Risk of Congenital Malformations

**DOI:** 10.1001/jamanetworkopen.2022.50366

**Published:** 2023-01-10

**Authors:** Ahhyung Choi, Yunha Noh, Han Eol Jeong, Eun-Young Choi, Kenneth K. C. Man, Jung Yeol Han, Hyun-Soo Kim, Dong Keon Yon, Ju-Young Shin

**Affiliations:** 1 School of Pharmacy, Sungkyunkwan University, Suwon, South Korea; 2 Department of Biohealth Regulatory Science, Sungkyunkwan University, Suwon, South Korea; 3 Research Department of Practice and Policy, University College London School of Pharmacy, London, United Kingdom; 4 Centre for Medicines Optimisation Research and Education, University College London Hospitals NHS Foundation Trust, London, United Kingdom; 5 Centre for Safe Medication Practice and Research, Department of Pharmacology and Pharmacy, Li Ka Shing Faculty of Medicine, University of Hong Kong, Hong Kong; 6 Korean Mothersafe Counselling Center, Department of Obstetrics and Gynecology, Inje University Ilsan Paik Hospital, Goyang, South Korea; 7 Division of Gastroenterology, Department of Internal Medicine, Yonsei University Wonju College of Medicine, Wonju, South Korea; 8 Center for Digital Health, Medical Science Research Institute, Kyung Hee University Medical Center, Kyung Hee University College of Medicine, Seoul, South Korea; 9 Department of Clinical Research Design and Evaluation, Samsung Advanced Institute for Health Sciences and Technology, Sungkyunkwan University, Seoul, South Korea

## Abstract

**Importance:**

Proton pump inhibitors (PPIs) are increasingly used during pregnancy; however, several observational studies have raised concerns about an increased risk of specific types of congenital malformations.

**Objective:**

To examine the association between PPI exposure during early pregnancy and the risk of congenital malformations.

**Design, Setting, and Participants:**

This population-based cohort study used data from the National Health Insurance Service–National Health Information Database of South Korea (2010-2020); sibling-controlled analyses were conducted to account for familial factors. A total of 2 696 216 pregnancies in women aged 19 to 44 years between June 1, 2011, and December 31, 2019, and their live-born infants were identified. Pregnant women who were exposed to known teratogens or who delivered infants with chromosomal abnormalities or genetic syndromes were excluded. Data on participant race and ethnicity were not collected because the National Health Information Database does not report this information.

**Exposures:**

Proton pump inhibitor use during the first trimester.

**Main Outcomes and Measures:**

Primary outcomes were major congenital malformations, congenital heart defects, cleft palate, hydrocephalus, and hypospadias. The subtypes of major congenital malformations and congenital heart defects were evaluated as exploratory outcomes. Propensity score fine stratification was used to control for potential confounders, and a weighted generalized linear model was used to estimate relative risks with 95% CIs.

**Results:**

Of 2 696 216 pregnancies (mean [SD] maternal age, 32.1 [4.2] years), 40 540 (1.5%; mean [SD] age, 32.4 [4.6] years) were exposed to PPIs during the first trimester. The absolute risk of major congenital malformations was 396.7 per 10 000 infants in PPI-exposed pregnancies and 323.4 per 10 000 infants in unexposed pregnancies. The propensity score–adjusted relative risks were 1.07 (95% CI, 1.02-1.13) for major congenital malformations, 1.09 (95% CI, 1.01-1.17) for congenital heart defects, 1.02 (95% CI, 0.72-1.43) for cleft palate, 0.94 (95% CI, 0.54-1.63) for hydrocephalus, and 0.77 (95% CI, 0.51-1.17) for hypospadias. In the sibling-controlled analyses, no associations were observed between PPI use and primary outcomes, including major congenital malformations (odds ratio, 1.05; 95% CI, 0.91-1.22) and congenital heart defects (odds ratio, 1.07; 95% CI, 0.88-1.30). A range of sensitivity analyses revealed results that were similar to the main findings.

**Conclusions and Relevance:**

In this cohort study, the use of PPIs during early pregnancy was not associated with a substantial increase in the risk of congenital malformations, although small increased risks were observed for major congenital malformations and congenital heart defects; findings from sibling-controlled analyses revealed that PPIs were unlikely to be major teratogens. These findings may help guide clinicians and patients in decision-making about PPI use in the first trimester.

## Key Points

**Question:**

Is proton pump inhibitor (PPI) use during pregnancy associated with an increased risk of congenital malformations?

**Findings:**

In this cohort study including 2 696 216 pregnancies in South Korea from 2011 to 2019, PPI use during the first trimester was not associated with a substantial increase in the risk of major congenital malformations, congenital heart defects, cleft palate, hydrocephalus, and hypospadias. Findings from the sibling-controlled analyses also revealed that PPIs were unlikely to be a major teratogen.

**Meaning:**

These findings may help guide clinicians and patients in decision-making about PPI use in early pregnancy.

## Teaser

This cohort study uses data from a nationwide database in South Korea to examine the association between exposure to proton pump inhibitors in the first trimester of pregnancy and the risk of congenital malformations among live-born infants.

## Introduction

Gastroesophageal reflux disease (GERD) is common during pregnancy, occurring in up to 80% of the pregnant population.^1^ Although mild symptoms can be alleviated by lifestyle modifications, acid-suppressive medications are often required for a substantial number of patients to manage their inadequately controlled symptoms. Specifically, the use of proton pump inhibitors (PPIs) during pregnancy has increased worldwide, which may be in part due to their acid-suppressing effects.^2,3^

However, despite the broad use of PPIs, the available evidence on their safety during pregnancy remains inconsistent. While earlier studies reported no associations between PPI use and major congenital malformations,^4,5,6,7^ several studies published within the past decade have found increased risk, particularly for congenital heart defects, cleft palate, hydrocephalus, and hypospadias.^8,9,10,11^ Accordingly, the latest meta-analysis, which included the previous studies,^4,5,6,7,8,9,10,11^ reported that PPI use during pregnancy was associated with a 28% increase in the risk of overall malformations.^12^ However, with respect to the quality of evidence, considerable uncertainties remain because previous studies had important methodological limitations, such as small sample size, inadequate adjustment of confounders, recall bias from self-reports, and exposure misclassification bias (eg, PPIs are available over the counter in many countries). Moreover, to our knowledge, no existing studies have accounted for genetic or familial factors in the association between prenatal PPI exposure and congenital malformations.

Given this conflicting evidence and the knowledge gaps among previous studies, the decision for clinicians and pregnant women to use PPIs during pregnancy remains in a gray area. Thus, we used the large-scale nationwide health care database of South Korea to investigate the association between PPI use during the first trimester and the risk of congenital malformations by conducting a population-based cohort study, complemented with sibling-controlled analyses to account for familial factors. We specifically focused on the risks of major congenital malformations, congenital heart defects, cleft palate, hydrocephalus, and hypospadias in view of conflicting evidence on the association of these risks with PPIs^6,7,8,9,10,11,12,13^ (eTable 1 in Supplement 1).

## Methods

### Data Source and Study Cohort

The study protocol was approved by the institutional review board of Sungkyunkwan University, and the need for informed patient consent was waived because our study used deidentified claims data. This study followed the Strengthening the Reporting of Observational Studies in Epidemiology (STROBE) reporting guideline for cohort studies.^14^

We conducted this nationwide cohort study using data from the National Health Insurance Service–National Health Information Database of South Korea (2010-2020), which contains longitudinal health care records of more than 50 million inhabitants (approximately 99% of the South Korean population).^15^ This database contains anonymized patient identifiers linked to sociodemographic data, data on inpatient and outpatient health care use (including diagnosis and prescription information), and health examination records. The health examination data contain records of mothers (eg, body mass index [BMI; calculated as weight in kilograms divided by height in meters squared] and smoking status) and their infants (eg, birth weight). A deterministic mother-child link, which was built based on the unique insurance identification number shared by family members,^16,17^ was provided by the National Health Insurance Service to identify all pregnancies resulting in live births between June 1, 2011, and December 31, 2019. The start of pregnancy was estimated based on a previously validated algorithm using administrative databases.^18^

For the study cohort, we first identified women aged 19 to 44 years at delivery. We then excluded pregnant women who were exposed to known teratogens or who delivered infants with chromosomal abnormalities, genetic syndromes, or malformation syndromes with known causes (eTable 2 in Supplement 1).

### Proton Pump Inhibitor Exposure

Exposure was defined as 1 or more prescriptions for PPIs during the first trimester (defined as the start of pregnancy to the 90th day of gestation), which is the period of embryogenesis. In South Korea, 7 PPIs (omeprazole, esomeprazole, lansoprazole, dexlansoprazole, pantoprazole, rabeprazole, and ilaprazole) are available only with a prescription, and no over-the-counter PPIs are available. The reference group consisted of pregnant women with no filled PPI prescriptions from 90 days before pregnancy through the end of the first trimester. Pregnant women who were exposed to PPIs from 90 days before the start of pregnancy but not during the first trimester were excluded to minimize misclassification.

### Outcomes

The primary outcomes were major congenital malformations, congenital heart defects, cleft palate, hydrocephalus, and hypospadias based on the findings of previous studies^8,9,10,11,12^ that have reported a potential increased risk associated with PPIs (eTable 1 in Supplement 1). The subtypes of major congenital malformations and congenital heart defects were also evaluated as exploratory outcomes only. The presence of congenital malformations was identified via infants’ records within the first year of life, and major congenital malformations and their subtypes were defined based on the European Surveillance of Congenital Anomalies (EUROCAT) classification system; any minor defects were excluded according to the EUROCAT exclusion list^19^ (eTable 2 in Supplement 1). The detailed definitions of outcomes are presented in eAppendix 1 in Supplement 1.

### Covariates

A propensity score (PS) was estimated for PPI-exposed pregnancies vs PPI-unexposed pregnancies using logistic regression analysis by including a broad range of covariates or potential confounders as independent variables. We included maternal demographic characteristics (eg, age and income level at delivery), parity and multiple gestations, indications for PPIs (eg, GERD and duodenitis), maternal medical conditions (eg, anxiety, diabetes, and epilepsy), medication use (eg, opioid analgesic medications and nonsteroidal anti-inflammatory drugs [NSAIDs]), obstetric comorbidity index,^20,21^ and measures of health care use (eg, number of outpatient visits, emergency department visits, and hospitalizations) (eTable 2 in Supplement 1). Data on participant race and ethnicity were not collected because the National Health Information Database does not report this information.

### Sibling Analyses

To account for potential confounding from family-related factors, we also performed sibling-controlled analyses. In these analyses, the risk of experiencing a study outcome was estimated among siblings; thus, shared familial and genetic factors within the family could be adjusted by comparing infants born to the same mother. A stratified logistic regression model was used, and only sibling pairs with discordant exposure and outcome status contributed to the estimates.^22^ Additional details on the sibling analyses, including the assumption test for carryover effects, are available in eAppendix 2 in Supplement 1.^23^

### Subgroup and Sensitivity Analyses

As a subgroup analysis, we assessed the prevalence of prenatal PPI use and investigated the association between the most frequently prescribed PPIs and the risk of experiencing study outcomes. To examine the dose-response associations, we further categorized the exposure groups by cumulative defined daily dose (DDD) during the first trimester as those using a cumulative DDD of less than 7, those using a cumulative DDD of 7 or more to less than 14, and those using a cumulative DDD of 14 or more.^24^

We conducted several sensitivity analyses to evaluate the robustness of our primary findings. First, we used pregnancies exposed to histamine 2 receptor antagonists as an active comparator instead of PPI-unexposed pregnancies. Second, we used the PPI discontinuers (those who used PPIs before pregnancy but discontinued use during the first trimester) as the reference group. Third, to address potential exposure misclassification, we redefined exposure as 2 or more prescriptions for PPIs. Fourth, we redefined the exposure assessment window as the fourth to tenth week of the gestational period, which is the duration known to be the most susceptible period of organogenesis. Fifth, we conducted a negative control analysis by redefining the exposure window as 5 to 8 months before pregnancy. A null association observed in this setting indirectly suggested that the findings of our main analysis were unlikely to be due to residual confounding. Sixth, to address potential confounding by indication, we restricted the study cohort to those with indications for PPIs. Seventh, we restricted the cohort to the first pregnancy episode to account for associations within women who had multiple pregnancies during our study period. Eighth, we restricted the cohort to singleton pregnancies to eliminate potential confounding from multiple gestations. Ninth, we restricted the study cohort to those who received health screening examinations to assess the potential of residual confounding from BMI and smoking status. Tenth, because our study only included pregnancies ending in live births, we evaluated the potential consequences of excluding pregnancies that were terminated (eAppendix 3, eTable 10 in Supplement 1).

### Statistical Analysis

We used standardized mean differences to compare the baseline characteristics of PPI-exposed and PPI-unexposed pregnancies; a value less than 0.1 indicated a balance in characteristics between the 2 groups. We calculated absolute risks and risk differences per 10 000 infants and relative risks (RRs) with 95% CIs. We used the PS fine stratification weighting method, which was reported to be efficient in controlling confounders at low exposure prevalence.^25^ After excluding pregnancies that had PS belonging to nonoverlapping regions of the whole PS distribution, we created 50 strata on the basis of the PS distribution of the pregnancies exposed to PPIs. After stratification, weights for the reference group were calculated using the distribution of the exposed group in each stratum to estimate the average treatment effect among the treated population. We then estimated the adjusted RRs with 95% CIs using generalized linear regression models, and we used a robust SE to account for correlations among women with multiple pregnancies. All analyses were performed using SAS Enterprise Guide, version 7.1 (SAS Institute Inc). Statistical significance was set at 2-sided *P* < .05 (with 95% CIs not overlapping 1.0).

## Results

### Study Cohort

The study cohort included 2 696 216 pregnancies (mean [SD] maternal age, 32.1 [4.2] years); of those, 40 540 women (1.5%; mean [SD] age, 32.4 [4.6] years) were prescribed PPIs during the first trimester (eFigure 1 in Supplement 1). Compared with women who were not exposed to PPIs during pregnancy, women who were exposed to PPIs were more likely to have indications for PPIs (eg, GERD), comorbid conditions (such as migraine and nausea and vomiting), and prescriptions for antidepressant, opioid, and NSAID medications. The overall burden of disease and health care use was higher among the PPI-exposed group compared with the PPI-unexposed group. After PS weighting, the 2 groups were well balanced on all characteristics, with standardized mean differences less than 0.10 (Table). Because the risk of hypospadias was estimated among women who delivered boys, we also presented the baseline characteristics of those pregnancies in eTable 3 in Supplement 1.

**Table.** Baseline Characteristics of the Study Cohort

Characteristic	Unadjusted			Propensity score–adjusted		
	Pregnancies, No. (%)		Standardized difference	Pregnancies, No. (%)		Standardized difference
	Exposed to PPI (n = 40 540)	Not exposed to PPI (n = 2 655 676)		Exposed to PPI (n = 40 537)	Not exposed to PPI (n = 2 655 675)	
Age, y						
Mean (SD)	32.4 (4.6)	32.1 (4.2)	0.07	32.4 (4.6)	32.4 (4.5)	0.01
Group						
19-25	2940 (7.3)	168 015 (6.3)	0.04	2939 (7.3)	189 416 (7.1)	0.01
26-30	10 283 (25.4)	716 715 (27.0)	−0.04	10 283 (25.4)	678 117 (25.5)	0
31-35	17 099 (42.2)	1 236 759 (46.6)	−0.09	17 097 (42.2)	1 129 307 (42.5)	−0.01
36-40	8705 (21.5)	478 324 (18.0)	0.09	8705 (21.5)	563 829 (21.2)	0.01
41-44	1513 (3.7)	55 863 (2.1)	0.10	1513 (3.7)	95 006 (3.6)	0.01
Medical aid recipient	551 (1.4)	14 089 (0.5)	0.09	550 (1.4)	33 465 (1.3)	0.01
Income level quartile						
1st (Lowest)	8633 (21.3)	512 266 (19.3)	0.05	8631 (21.3)	561 694 (21.2)	0
2nd	9941 (24.5)	636 869 (24.0)	0.01	9940 (24.5)	647 928 (24.4)	0
3rd	13 524 (33.4)	932 367 (35.1)	−0.04	13 524 (33.4)	890 261 (33.5)	0
4th (Highest)	8442 (20.8)	574 174 (21.6)	−0.02	8442 (20.8)	555 792 (20.9)	0
Region						
Metropolitan	27 404 (67.6)	1 856 731 (69.9)	−0.05	27 401 (67.6)	1 795 500 (67.6)	0
Rural	1 (<0.1)	1244 (<0.1)	−0.03	1 (<0.1)	76 (<0.1)	0
Urban	13 135 (32.4)	797 701 (30.0)	0.05	13 135 (32.4)	860 099 (32.4)	0
Nulliparity	18 894 (46.6)	1 353 486 (51.0)	−0.09	18 893 (46.6)	1 243 018 (46.8)	0
Multiple gestation	803 (2.0)	49 465 (1.9)	0.01	803 (2.0)	53 005 (2.0)	0
Year of delivery						
2011	1912 (4.7)	217 216 (8.2)	−0.14	1912 (4.7)	126 365 (4.8)	0
2012	3939 (9.7)	392 469 (14.8)	−0.16	3939 (9.7)	257 197 (9.7)	0
2013	3998 (9.9)	348 818 (13.1)	−0.10	3998 (9.9)	260 767 (9.8)	0
2014	4241 (10.5)	345 869 (13.0)	−0.08	4241 (10.5)	276 408 (10.4)	0
2015	5237 (12.9)	340 907 (12.8)	0	5237 (12.9)	341 690 (12.9)	0
2016	5194 (12.8)	311 678 (11.7)	0.03	5192 (12.8)	340 911 (12.8)	0
2017	5554 (13.7)	264 578 (10.0)	0.12	5553 (13.7)	364 151 (13.7)	0
2018	5358 (13.2)	231 823 (8.7)	0.14	5358 (13.2)	352 483 (13.3)	0
2019	5107 (12.6)	202 318 (7.6)	0.17	5107 (12.6)	335 703 (12.6)	0
Indications						
GERD	28 299 (69.8)	145 292 (5.5)	1.78	28 292 (69.8)	1 856 145 (69.9)	0
Barrett esophagus	11 (<0.1)	29 (<0.1)	0.02	11 (<0.1)	455 (<0.1)	0.01
Ulcer	7282 (18.0)	85 865 (3.2)	0.49	7279 (18.0)	450 490 (17.0)	0.03
Gastritis and duodenitis	27 773 (68.5)	795 236 (29.9)	0.84	27 771 (68.5)	1 849 390 (69.6)	−0.02
Dyspepsia	6941 (17.1)	208 036 (7.8)	0.28	6940 (17.1)	444 976 (16.8)	0.01
Heartburn	1831 (4.5)	61 219 (2.3)	0.12	1830 (4.5)	118 416 (4.5)	0
Zollinger-Ellison syndrome	2 (<0.1)	19 (<0.1)	0.01	2 (<0.1)	161 (<0.1)	0
*Helicobacter pylori* infection	105 (0.3)	366 (<0.1)	0.07	104 (0.3)	5268 (0.2)	0.01
Medical conditions						
Anxiety	1360 (3.4)	24 192 (0.9)	0.17	1359 (3.4)	83 867 (3.2)	0.01
Diabetes	530 (1.3)	17 762 (0.7)	0.07	530 (1.3)	34 106 (1.3)	0
Epilepsy	144 (0.4)	4587 (0.2)	0.04	144 (0.4)	8737 (0.3)	0
Headache (including migraine)	4202 (10.4)	130 177 (4.9)	0.21	4200 (10.4)	273 552 (10.3)	0
Hypertension	510 (1.3)	14 822 (0.6)	0.07	510 (1.3)	32 086 (1.2)	0.01
Kidney disease	249 (0.6)	7219 (0.3)	0.05	249 (0.6)	16 413 (0.6)	0
Alcohol or drug dependence	82 (0.2)	1806 (0.1)	0.04	82 (0.2)	5272 (0.2)	0
Tobacco dependence	3 (<0.1)	55 (<0.1)	0.01	3 (<0.1)	174 (<0.1)	0
Nausea and vomiting	9704 (23.9)	320 364 (12.1)	0.31	9701 (23.9)	637 280 (24.0)	0
Prescription drug use						
Antidepressants	2699 (6.7)	39 355 (1.5)	0.26	2698 (6.7)	163 102 (6.1)	0.02
Antidiabetics	401 (1.0)	14 492 (0.5)	0.05	401 (1.0)	26 302 (1.0)	0
Antihypertensives	1826 (4.5)	42 494 (1.6)	0.17	1826 (4.5)	114 731 (4.3)	0.01
Benzodiazepines	11 936 (29.4)	215 099 (8.1)	0.57	11 933 (29.4)	757 246 (28.5)	0.02
Corticosteroids	21 022 (51.9)	884 702 (33.3)	0.38	21 020 (51.9)	1 396 041 (52.6)	−0.01
Fertility	2194 (5.4)	164 028 (6.2)	−0.03	2194 (5.4)	147 122 (5.5)	−0.01
Opioid analgesics	24 455 (60.3)	1 046 484 (39.4)	0.43	24 452 (60.3)	1 625 380 (61.2)	−0.02
NSAIDs	31 194 (76.9)	1 547 249 (58.3)	0.41	31 191 (76.9)	2 076 544 (78.2)	−0.03
Thyroid hormones	1913 (4.7)	102 629 (3.9)	0.04	1913 (4.7)	126 222 (4.8)	0
Antithyroids	402 (1.0)	15 615 (0.6)	0.05	402 (1.0)	26 120 (1.0)	0
Lipid lowering	426 (1.1)	6599 (0.2)	0.10	426 (1.1)	25 383 (1.0)	0.01
Stimulants	2 (<0.1)	57 (<0.1)	0.01	2 (<0.1)	159 (<0.1)	0
Triptans	368 (0.9)	6345 (0.2)	0.09	366 (0.9)	22 732 (0.9)	0.01
Antiemetics	12 172 (30.0)	344 117 (13.0)	0.43	12 169 (30.0)	803 042 (30.2)	−0.01
Fluconazole	2619 (6.5)	102 430 (3.9)	0.12	2619 (6.5)	170 212 (6.4)	0
Obstetric comorbidity index score, mean (SD)	0.7 (1.0)	0.5 (0.8)	0.21	0.7 (1.0)	0.7 (1.0)	0.01
No. of outpatient visits, mean (SD)	8.7 (9.0)	5.3 (5.6)	0.45	8.7 (8.9)	8.7 (7.0)	0
No. of emergency department visits, mean (SD)	0.2 (1.0)	0.1 (0.4)	0.11	0.2 (0.6)	0.2 (0.5)	−0.01
No. of hospitalizations, mean (SD)	0.1 (0.5)	0.1 (0.3)	0.13	0.1 (0.5)	0.1 (0.4)	0

Abbreviations: GERD, gastroesophageal reflux disease; NSAID, nonsteroidal anti-inflammatory drug; PPI, proton pump inhibitor.

### Risk of Congenital Malformations

**Figure 1.**
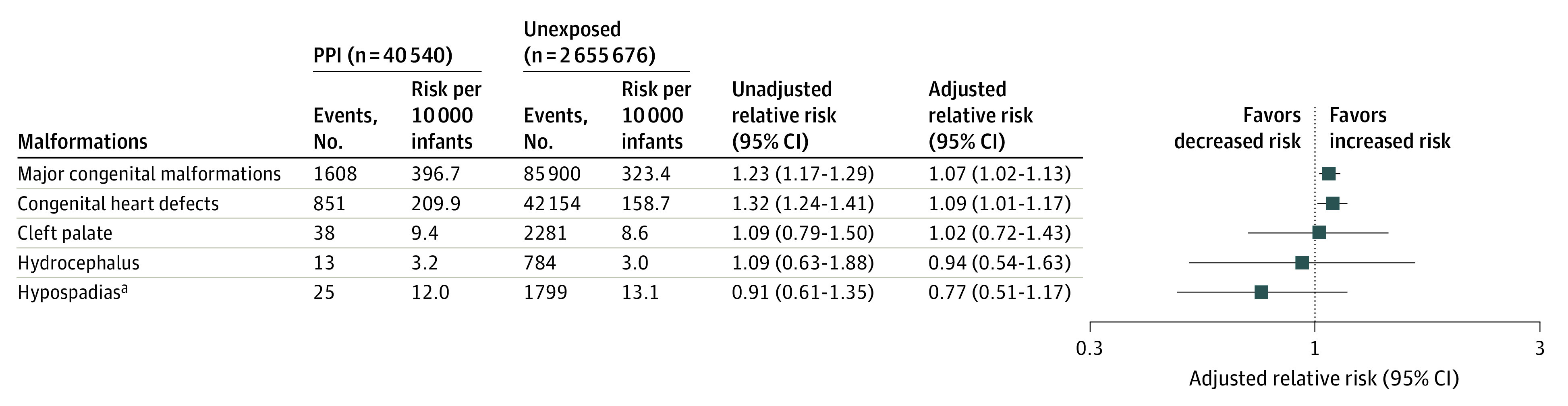
Association Between Proton Pump Inhibitor (PPI) Exposure in Pregnancy and Risk of Congenital Malformations ^a^Estimated among 20 900 pregnant women exposed to PPIs and 1 371 387 pregnant women not exposed to PPIs who delivered boys.

The absolute risk of experiencing primary outcomes among PPI-exposed and PPI-unexposed pregnancies as well as the unadjusted and adjusted RR estimates are shown in Figure 1. The absolute risk of major congenital malformations was 396.7 per 10 000 infants in PPI-exposed pregnancies and 323.4 per 10 000 infants in PPI-unexposed pregnancies. Before adjusting the baseline covariates, PPI-exposed pregnancies had a higher risk of major congenital malformations (RR, 1.23; 95% CI, 1.17-1.29) and congenital heart defects (RR, 1.32; 95% CI, 1.24-1.41). After PS adjustment, the risk estimates were attenuated for major congenital malformations (RR, 1.07; 95% CI, 1.02-1.13), congenital heart defects (RR, 1.09; 95% CI, 1.01-1.17), cleft palate (RR, 1.02; 95% CI, 0.72-1.43), hydrocephalus (RR, 0.94; 95% CI, 0.54-1.63), and hypospadias (RR, 0.77; 95% CI, 0.51-1.17). Unadjusted and adjusted risk differences per 10 000 infants with 95% CIs for primary outcomes are reported in eTable 4 in Supplement 1.

**Figure 2.**
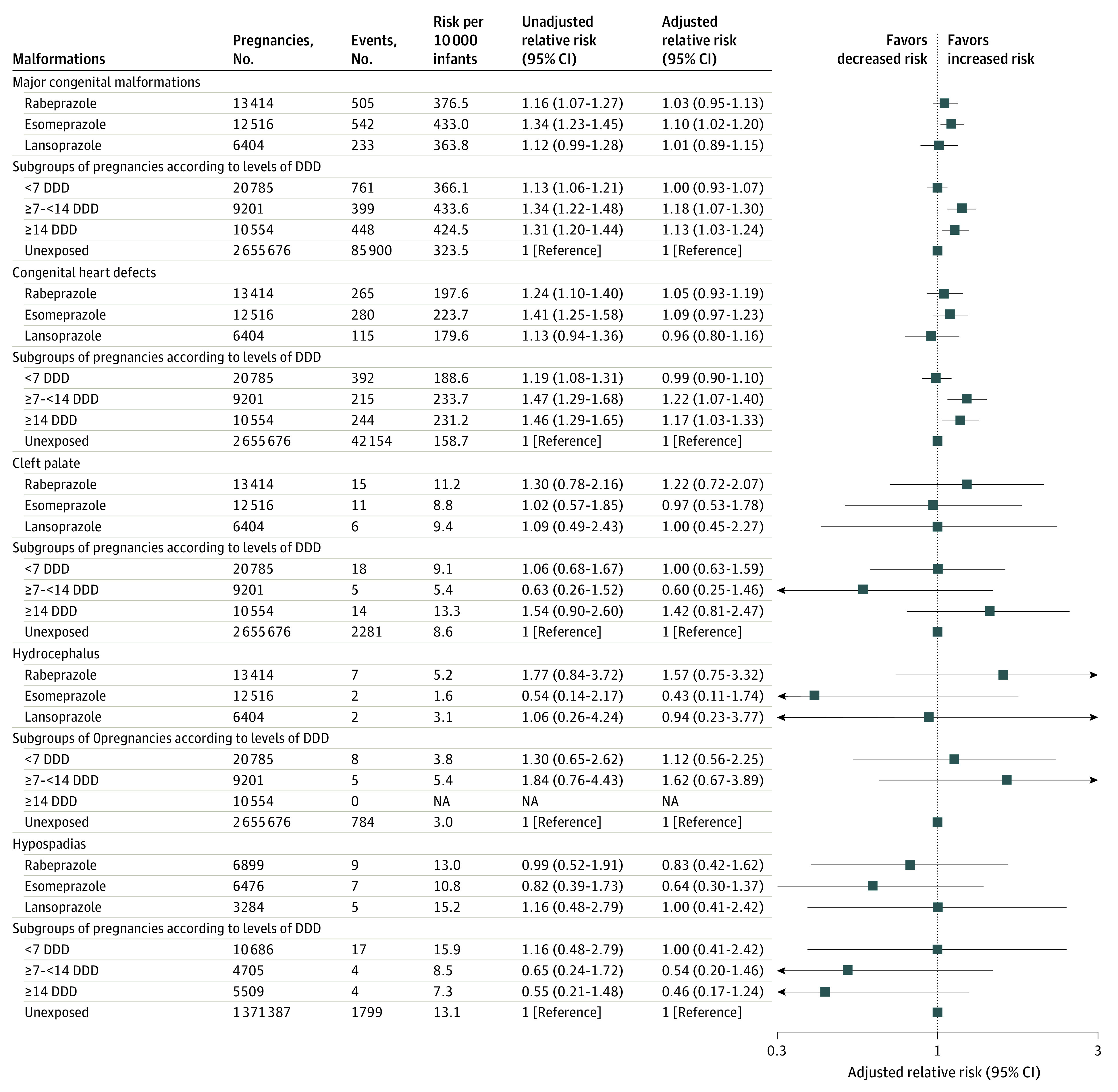
Subgroup Analysis of Association Between Proton Pump Inhibitor Exposure in Pregnancy and Risk of Congenital Malformations DDD indicates defined daily dose; and NA, not applicable.

The prevalence of PPI use during pregnancy increased from 1.01% in 2012 to 2.61% in 2019. The most frequently prescribed PPI was rabeprazole, followed by esomeprazole and lansoprazole (eFigure 2 in Supplement 1). No associations were observed between individual PPIs and each primary outcome, except for esomeprazole, which was associated with a small increase in the risk of major congenital malformations (RR, 1.10; 95% CI, 1.02-1.20) (Figure 2). There was a modest increase in the risk of major congenital malformations and congenital heart defects in the group with a cumulative DDD of 7 or more to less than 14 and in the group with a cumulative DDD of 14 or more (Figure 2).

As exploratory analyses, we evaluated the risk of subtypes of major congenital malformations and congenital heart defects (eTables 5-7 in Supplement 1). Elevated risks were observed for respiratory system defects (adjusted RR, 1.77; 95% CI, 1.12-2.79) and abdominal wall defects (adjusted RR, 2.74; 95% CI, 1.14-6.57), which corresponded to adjusted risk differences of 2.68 (95% CI, 0.14-5.23) per 10 000 infants for respiratory system defects and 0.94 (95% CI, −0.26 to 2.14) per 10 000 infants for abdominal wall defects.

### Sibling Analyses

**Figure 3.**
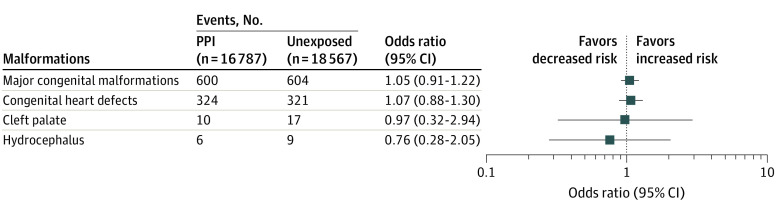
Sibling Analysis of Association Between Proton Pump Inhibitor (PPI) Exposure in Pregnancy and Risk of Congenital Malformations

In the sibling analyses, we identified 16 730 families with siblings of infants discordant for PPI exposure. The risk of major congenital malformations and congenital heart defects was attenuated and included the null in these analyses, with adjusted odds ratios (ORs) of 1.05 (95% CI, 0.91-1.22) for major congenital malformations and 1.07 (95% CI, 0.88-1.30) for congenital heart defects (Figure 3). Additional results from the sibling analyses are described in eAppendix 2, eTables 8 and 9 in Supplement 1.

### Sensitivity Analyses

**Figure 4.**
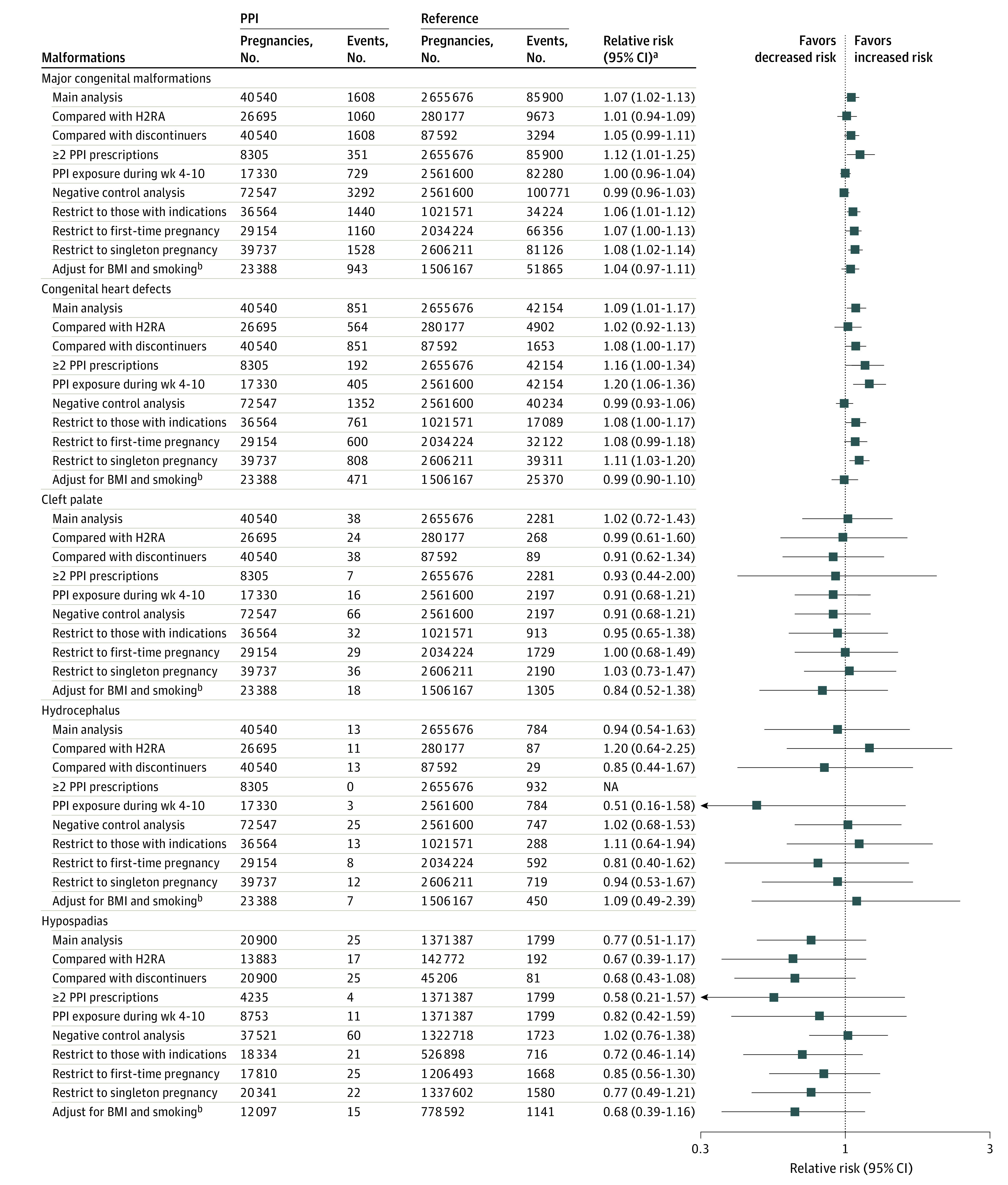
Sensitivity Analysis of Association Between Proton Pump Inhibitor (PPI) Exposure in Pregnancy and Risk of Congenital Malformations BMI indicates body mass index (calculated as weight in kilograms divided by height in meters squared); H2RA, histamine 2 receptor antagonist; and NA, not applicable. ^a^Propensity score–adjusted relative risk. ^b^Restricted to those who received a health screening examination and had information available on BMI and smoking status.

Overall, sensitivity analyses yielded estimates that were generally consistent with our main findings (Figure 4). When compared with those who were exposed to histamine 2 receptor antagonists or those who discontinued use of PPIs, no increased risks were observed for all primary outcomes. To account for residual confounding from BMI and smoking, additional analyses among those with BMI and smoking data were conducted, and the results were robust. We also quantified the potential consequences of including only pregnancies ending in live births in the study cohort (eAppendix 3 in Supplement 1). Under the most extreme scenario, which assumed the live birth probability of PPI-exposed infants with malformation was 35%, the RRs for major congenital malformations and congenital heart defects remained lower than 1.30 (1.26 for major congenital malformations and 1.28 for congenital heart defects) (eFigure 3 and eFigure 4 in Supplement 1).

## Discussion

### Principal Findings

In this large-scale nationwide cohort study that included 40 540 pregnancies exposed to PPIs among 2 696 216 pregnancies, PPI exposure during the first trimester was not associated with a substantially increased risk of major congenital malformations, congenital heart defects, cleft palate, hydrocephalus, and hypospadias. Although a significant association was found between PPI exposure and major congenital malformations (adjusted RR, 1.07; 95% CI, 1.02-1.13) and congenital heart defects (adjusted RR, 1.09; 95% CI, 1.01-1.17), these associations may not be deemed clinically meaningful considering the nature of observational studies and the magnitude of the point estimates. Likewise, attenuated and null estimates observed in the sibling-controlled analyses and the results from a range of sensitivity analyses further supported the finding that prenatal PPI exposure was not associated with a substantial increase in the risk of congenital malformations.

Teratogenic consequences of PPIs are biologically plausible because PPIs cross the placenta and can induce deficiencies in fetal morphogenesis.^26,27^ Some PPIs are also known to block specific transporters on the placenta that play a substantial role in protecting the fetus from toxic substances.^28,29,30^ There may also be potential indirect pathways. For example, PPIs have been associated with nutrient deficiency and may also be a source of exposure to phthalate coatings, which could increase the risk of specific malformations.^31,32,33,34,35,36^ However, theoretical mechanisms are not necessarily applicable to animals and humans, and preclinical studies did not suggest teratogenicity of PPIs, even at doses 56 times higher than that recommended for humans, although dose-related fetal deaths in pregnant rats and rabbits were reported.^37,38,39^ Likewise, the findings of the present study also suggested no meaningful increase in the risk of malformations.

The latest meta-analysis^12^ of 18 observational studies reported that PPI use during pregnancy was associated with a 28% increase in the risk of overall malformations. In particular, the risk was higher when based on the case-control studies (pooled OR, 2.04; 95% CI, 1.46-2.86) rather than the cohort studies (pooled OR, 1.12; 95% CI, 0.99-1.27).^12^ When interpreting these findings, potential concerns that exist in most studies reporting positive associations between PPI and specific malformations should be taken into consideration. These concerns include the possibility of residual confounding (eg, from underlying comorbidities and concomitant medications), lack of statistical power due to small study populations, and recall bias. Meanwhile, the current study addressed these shortcomings by including the largest study cohort to date (40 540 PPI-exposed pregnancies), carefully adjusting for numerous potential confounders, and conducting various sensitivity and sibling-controlled analyses, finding no association between PPI use and a substantial risk of malformations.

Moreover, the inclusion of a large sample and detailed data on medication use allowed us to evaluate the risk associated with individual PPIs along with dose-response associations. Earlier studies^4,5^ that excluded a large increase in the risk of malformations associated with maternal PPI use have generally focused on omeprazole based on dose-related mortality observed in animal studies.^37,39^ To our knowledge, only 1 study^6^ to date has investigated PPIs both as a class and as individual agents; that study included omeprazole and other PPIs, finding no associations with overall malformations. Our study, based on more recent data, further adds to the literature and provides evidence on the fetal safety of PPIs. In the dose-response analysis, we found potential dose-response associations for major congenital malformations and congenital heart defects, although the magnitude of the point estimates was modest. This result is in contrast to the findings of a previous study^7^ that found no association between PPIs and the risk of congenital malformations in terms of DDD. One possible explanation for the dose-response association observed in our study could be confounding by severity on the basis that PPIs are not only prescribed for the treatment of GERD but are also widely coprescribed with NSAIDs to prevent NSAID-associated gastric ulcers.^40^ For instance, patients using NSAIDs for prolonged periods to manage severe underlying conditions are likely to have been simultaneously exposed to higher cumulative PPIs, which in turn could have had implications for the slightly increased risk of major congenital malformations and congenital heart defects observed in pregnant women who received a cumulative DDD of 7 or more.

While there was no substantial increase in the risk of most congenital malformation subtypes in the exploratory analyses, we observed somewhat elevated risks of respiratory system defects (adjusted RR, 1.77; 95% CI, 1.12-2.79; risk difference, 2.68 per 10 000 infants) and abdominal wall defects (adjusted RR, 2.74; 95% CI, 1.14-6.57; risk difference, 0.94 per 10 000 infants). Nevertheless, these results should be interpreted with caution, given that these types of malformations were not previously reported and, to our knowledge, there are no clear pharmacological mechanisms supporting this observation. Thus, further replications in other populations are warranted. In the meantime, it is notable that the absolute risk of these malformations was fairly low.

### Strength and Limitations

This study has several strengths. Apart from being large scale, data from a nationwide longitudinal claims database allowed us to minimize the risk of selection and recall bias. Moreover, the rich individual-level health care data enabled us to characterize numerous potential confounders, including both inpatient and outpatient medication exposures and medical conditions. In addition, because PPIs are available only with prescriptions in South Korea, exposure misclassification owing to over-the-counter availability is unlikely in our study.

This study also has several limitations. First, exposure misclassification was still possible because having a prescription does not necessarily indicate the actual use or consumption of medications. To account for this limitation, we redefined the exposure as 2 or more PPI prescriptions to increase the specificity of the sensitivity analyses, which did not substantially change our results. Second, because the ascertainment of congenital malformations was based on diagnostic codes, outcome misclassification is possible. However, to increase specificity, we defined the outcomes by incorporating primary diagnosis codes and malformation-specific procedure codes to refer to the previous well-validated outcome definitions provided in other administrative data. Moreover, the additional analysis restricted to inpatient diagnosis yielded risk estimates similar to our main findings (eAppendix 1 in Supplement 1).

Third, as in any observational study, residual confounding cannot be ruled out. However, because our study observed null or close to null findings, if positive associations were present, these unmeasured confounders would have had an association with PPIs and would also have had preventive associations with outcomes, which is unlikely. Fourth, although we conducted sibling-controlled analyses to further account for genetic or familial factors, there is a potential risk of amplification of confounding by unmeasured confounders not shared by the siblings.^41,42^ Moreover, the measurement error in the exposure in the sibling comparison design may produce increased attenuation of the association.^41^

Fifth, although we estimated the start of pregnancy using an algorithm that was previously validated in administrative databases, misclassification of the exposure window may exist. Sixth, the study cohort only included pregnancies that resulted in live births and did not include terminated pregnancies owing to the unavailability of gestational ages for nonlive births. If PPI-exposed pregnancies had a higher proportion of fetuses with malformations that led to abortions or stillbirths, our estimates may be biased. Thus, we analyzed the potential consequences of such bias in sensitivity analyses, and the results revealed that even under the most extreme scenario, the risk was minimal. However, it should be noted that the consequences of restricting the analysis to live births among the sibling population may yield different results compared with the full population in the main analysis.

## Conclusions

Overall, this large nationwide cohort study of 2 696 216 pregnancies found that PPI use during the first trimester of pregnancy was not associated with a substantial increase in the risk of major congenital malformations, congenital heart defects, cleft palate, hydrocephalus, and hypospadias, although there were small increases in the risk of major congenital malformations and congenital heart defects; findings from the sibling-controlled analysis and a wide range of sensitivity analyses suggest that PPIs are unlikely to be a major teratogen. Given the increasing use of PPIs during pregnancy, our findings may help guide clinicians and patients in decision-making about the use of PPIs during the first trimester.

## Notes

Supplement 1. **eTable 1.** Previous Studies on Proton Pump Inhibitors (PPIs) and the Risk of Malformations **eTable 2.** Codes Used to Define Exclusion Criteria, Exposures, Outcomes of Interest, and Covariates **eTable 3.** Baseline Characteristics of Pregnant Women Who Delivered Boys for the Analysis of Hypospadias **eTable 4.** Risk Difference and 95% CIs Comparing the Risk of Congenital Malformations Between Pregnancies Exposed and Not Exposed to Proton Pump Inhibitors (PPIs) **eTable 5.** Association Between Proton Pump Inhibitor (PPI) Exposure in Pregnancy and the Risk of Subtypes of Major Congenital Malformations **eTable 6.** Relative Risks and 95% CIs Comparing the Risk of Subtypes of Congenital Heart Defects Between Pregnancies Exposed and Not Exposed to Proton Pump Inhibitors (PPIs) **eTable 7.** Risk Differences and 95% CIs Comparing the Risk of Subtypes of Congenital Heart Defects Between Pregnancies Exposed and Not Exposed to Proton Pump Inhibitors (PPIs) **eTable 8.** Association Between Proton Pump Inhibitor (PPI) Exposure in Pregnancy and the Risk of Congenital Malformations Among Sibling Populations **eTable 9.** Odds Ratios of Proton Pump Inhibitor (PPI) Exposure During the Second Pregnancy According to Congenital Malformation Status of the First Sibling **eFigure 1.** Study Flowchart **eFigure 2.** Frequency of Pregnancies Exposed to Proton Pump Inhibitors (PPIs) During the First Trimester **eAppendix 1.** Outcome Definition **eAppendix 2.** Additional Details on the Sibling Analysis **eAppendix 3.** Potential Consequences of Including Only Live Births **eTable 10.** Probability of Live Births **eFigure 3.** Corrected Relative Risk for the Association Between Proton Pump Inhibitor (PPI) Exposure During the First Trimester and Major Congenital Malformations **eFigure 4.** Corrected Relative Risk for the Association Between Proton Pump Inhibitor (PPI) Exposure During the First Trimester and Congenital Heart Defects

Supplement 2.
Data Sharing Statement
