# Supplementary material for: Association Between Proton Pump Inhibitor Use During Early Pregnancy and Risk of Congenital Malformations
Source: JAMA Netw Open. 2023 Jan 10;6(1):e2250366. doi: 10.1001/jamanetworkopen.2022.50366 (PMC9856708; doi:10.1001/jamanetworkopen.2022.50366)
Supplement: Supplement 1. — eTable 1. Previous Studies on Proton Pump Inhibitors (PPIs) and the Risk of Malformations eTable 2. Codes Used to Define Exclusion Criteria, Exposures, Outcomes of Interest, and Covariates eTable 3. Baseline Characteristics of Pregnant Women Who Delivered Boys for the Analysis of Hypospadias eTable 4. Risk Difference and 95% CIs Comparing the Risk of Congenital Malformations Between Pregnancies Exposed and Not Exposed to Proton Pump Inhibitors (PPIs) eTable 5. Association Between Proton Pump Inhibitor (PPI) Exposure in Pregnancy and the Risk of Subtypes of Major Congenital Malformations eTable 6. Relative Risks and 95% CIs Comparing the Risk of Subtypes of Congenital Heart Defects Between Pregnancies Exposed and Not Exposed to Proton Pump Inhibitors (PPIs) eTable 7. Risk Differences and 95% CIs Comparing the Risk of Subtypes of Congenital Heart Defects Between Pregnancies Exposed and Not Exposed to Proton Pump Inhibitors (PPIs) eTable 8. Association Between Proton Pump Inhibitor (PPI) Exposure in Pregnancy and the Risk of Congenital Malformations Among Sibling Populations eTable 9. Odds Ratios of Proton Pump Inhibitor (PPI) Exposure During the Second Pregnancy According to Congenital Malformation Status of the First Sibling eFigure 1. Study Flowchart eFigure 2. Frequency of Pregnancies Exposed to Proton Pump Inhibitors (PPIs) During the First Trimester eAppendix 1. Outcome Definition eAppendix 2. Additional Details on the Sibling Analysis eAppendix 3. Potential Consequences of Including Only Live Births eTable 10. Probability of Live Births eFigure 3. Corrected Relative Risk for the Association Between Proton Pump Inhibitor (PPI) Exposure During the First Trimester and Major Congenital Malformations eFigure 4. Corrected Relative Risk for the Association Between Proton Pump Inhibitor (PPI) Exposure During the First Trimester and Congenital Heart Defects [file jamanetwopen-e2250366-s001.pdf]

Supplemental Online Content

Choi A, Noh Y, Jeong HE, et al. Association between proton pump inhibitor use during early pregnancy and risk of congenital malformations. *JAMA Netw Open*. 2023;6(1):e2250366. doi:10.1001/jamanetworkopen.2022.50366

eTable 1. Previous Studies on Proton Pump Inhibitors (PPIs) and the Risk of Malformations

eTable 2. Codes Used to Define Exclusion Criteria, Exposures, Outcomes of Interest, and Covariates

eTable 3. Baseline Characteristics of Pregnant Women Who Delivered Boys for the Analysis of Hypospadias

eTable 4. Risk Difference and 95% CIs Comparing the Risk of Congenital Malformations Between Pregnancies Exposed and Not Exposed to Proton Pump Inhibitors (PPIs)

eTable 5. Association Between Proton Pump Inhibitor (PPI) Exposure in Pregnancy and the Risk of Subtypes of Major Congenital Malformations

eTable 6. Relative Risks and 95% CIs Comparing the Risk of Subtypes of Congenital Heart Defects Between Pregnancies Exposed and Not Exposed to Proton Pump Inhibitors (PPIs)

eTable 7. Risk Differences and 95% CIs Comparing the Risk of Subtypes of Congenital Heart Defects Between Pregnancies Exposed and Not Exposed to Proton Pump Inhibitors (PPIs)

eTable 8. Association Between Proton Pump Inhibitor (PPI) Exposure in Pregnancy and the Risk of Congenital Malformations Among Sibling Populations

eTable 9. Odds Ratios of Proton Pump Inhibitor (PPI) Exposure During the Second Pregnancy According to Congenital Malformation Status of the First Sibling

eFigure 1. Study Flowchart

eFigure 2. Frequency of Pregnancies Exposed to Proton Pump Inhibitors (PPIs) During the First Trimester

eAppendix 1. Outcome Definition

eAppendix 2. Additional Details on the Sibling Analysis

eAppendix 3. Potential Consequences of Including Only Live Births

eTable 10. Probability of Live Births

eFigure 3. Corrected Relative Risk for the Association Between Proton Pump Inhibitor (PPI) Exposure During the First Trimester and Major Congenital Malformations

eFigure 4. Corrected Relative Risk for the Association Between Proton Pump Inhibitor (PPI) Exposure During the First Trimester and Congenital Heart Defects

This supplemental material has been provided by the authors to give readers additional information about their work.

© 2023 Choi A et al. *JAMA Network Open*.

eTable 1. Previous Studies on Proton Pump Inhibitors (PPIs) and the Risk of Malformations

Study	Data Source	Number of study population	Outcome	OR/RR (95% CI)	Note
Rhim et al, 2010	THIN database	Total: 21,975	Cardiac defects	2.14 (1.37-3.35)	• Abstract only
Pasternak et al, 2010	Medical Birth Register, 1996-2008	Exposed: 3,651<br>Unexposed: 837,317	Major birth defects<br>Hypospadias	1.10 (0.91-1.34)<br>1.55 (0.89-2.70)	
Anderka et al, 2012	National Birth Defects Prevention Study (NBDPS)	Exposed case: 5 (cleft palate), 7 (hypospadias)	Cleft palate<br>Hypospadias	2.96 (1.05-8.32)<br>4.36 (1.21-15.81)	• Recall bias<br>• Limited sample size
Matok et al, 2012	Clalit database	Exposed: 1,186<br>Unexposed: 109,579	Major congenital malformations<br>Cardiovascular malformations<br>Atrial septal defect	1.06 (0.84-1.33)<br>0.98 (0.70-1.38)<br>1.44 (0.88-2.34)	
Lind et al, 2013	National Birth Defects Prevention Study (NBDPS)	Exposed case: 6 (lansoprazole), 5 (omeprazole)<br>Total case: 1,537	Hypospadias	2.82 (0.91-8.76)<br>(Omeprazole: 2.9 (0.7-11.3), lansoprazole: 2.8 (0.8-9.8))	• Limited sample size<br>• Only included omeprazole and lansoprazole
Munch et al, 2014	Danish national birth register	Exposed: 7,762<br>Unexposed: 844,721	Hydrocephalus	2.35 (1.26-4.41)	• Only adjusted for age, parity, multiple births, year of birth, and child's sex
Erichsen et al, 2014	Danish nationwide registries, 1997-2009	Exposed: 2,926<br>Unexposed: 427,643	Hypospadias	1.4 (0.9-2.3)	

© 2023 Choi A et al. *JAMA Network Open*.

Li et al, 2020	Meta-analysis of 18 studies	Overall malformation, pooled estimate: 1.28 (1.09-1.52)<br>• Pooled estimate for case-control studies (N=6): 2.04 (1.46-2.86)<br>• Pooled estimate for cohort studies (N=12): 1.12 (0.99-1.27)

Abbreviations: PPI=Proton Pump Inhibitor, OR=Odds Ratio, RR=Relative Risk, CI=Confidence Interval

References

1. Rhim AD, Hardy JR, Haynes K, Testani JM, Yang YX. Maternal use of proton pump inhibitors (PPI) during pregnancy is associated with an increased risk for cardiac birth defects: analysis of 208,951 pregnancies from the GPRD/THIN database. Abstract 475b. AGA Abstracts; 2010:S-63. Accessed October 1, 2021. https://www.academia.edu/23321167/475b_Maternal_use_of_Proton_Pump_Inhibitors_PPI_During_Pregnancy_is_Associated_With_an_Increased_Risk_for_Cardiac_Birth_Defects_Analysis_of_208_951_Pregnancies_From_the_GPRD_THIN_Database

2. Pasternak B, Hviid A. Use of proton-pump inhibitors in early pregnancy and the risk of birth defects. N Engl J Med. 2010;363(22):2114-2123. Medline:21105793 doi:10.1056/NEJMoa1002689

3. Anderka M, Mitchell AA, Louik C, Werler MM, Hernández-Diaz S, Rasmussen SA; National Birth Defects Prevention Study. Medications used to treat nausea and vomiting of pregnancy and the risk of selected birth defects. Birth Defects Res A Clin Mol Teratol. 2012;94(1):22-30. Medline:22102545 doi:10.1002/bdra.22865

4. Matok I, Levy A, Wiznitzer A, Uziel E, Koren G, Gorodischer R. The safety of fetal exposure to proton-pump inhibitors during pregnancy. Dig Dis Sci. 2012;57(3):699-705. Medline:22038541 doi:10.1007/s10620-011-1940-3

5. Lind JN, Tinker SC, Broussard CS, et al; National Birth Defects Prevention Study. Maternal medication and herbal use and risk for hypospadias: data from the National Birth Defects Prevention Study, 1997-2007. Pharmacoepidemiol Drug Saf. 2013;22(7):783-793. Medline:23620412 doi:10.1002/pds.3448

6. Munch TN, Rasmussen MLH, Wohlfahrt J, Juhler M, Melbye M. Risk factors for congenital hydrocephalus: a nationwide, register-based, cohort study. J Neurol Neurosurg Psychiatry. 2014;85(11):1253-1259. Medline:24667207 doi:10.1136/jnnp-2013-306941

7. Li CM, Zhernakova A, Engstrand L, Wijmenga C, Brusselaers N. Systematic review with meta-analysis: the risks of proton pump inhibitors during pregnancy. Aliment Pharmacol Ther. 2020;51(4):410-420. Medline:31909512 doi:10.1111/apt.15610

© 2023 Choi A et al. *JAMA Network Open*.

eTable 2. Codes Used to Define Exclusion Criteria, Exposures, Outcomes of Interest, and Covariates

Categories	Codes
**Exclusion criteria**	**ICD-10 or ATC codes**
Teratogenic/genetic syndromes, microdeletions, chromosomal abnormalities, and malformation syndromes with known causes	D821, P350-P351, P371, Q447, Q619, Q751, Q754, Q771-Q772, Q780, Q796, Q85-Q87, Q90-Q93, Q95-Q99
Known or potential teratogens	Antineoplastic agent (L01), warfarin (B01AA03), lithium (N05AN), systemic retinoids (D10BA, D05BB), misoprostol (A02BB01, G02AD06, M01AE56), thalidomide (L04AX02, L04AX04, L04AX06), androgens (G03B, G03E, G03XA), tetracycline derivative (J01AA), carbamazepine (N03AF01), oxcarbazepine (N03AF02), phenobarbital (N03AA02), phenytoin (N03AB02), valproic acid (N03AG01)
**Exposures**	**ATC codes**
Proton Pump Inhibitors	A02BC
**Outcomes of interest**	**ICD-10 codes**
Major congenital malformations	
Nervous system	Q00-Q07
Hydrocephalus	Q03
Eye	Q100, Q104, Q106-Q109, Q11-Q12, Q130-Q134, Q136-Q139, Q14-Q15
Ear, face, and neck	Q16, Q176-Q178, Q183, Q188
Heart defects	Defects of cardiac chambers and their connections: Q20<br>Septal defects: Q21 (Ventricular, VSD: Q210, atrial (ASD): Q211, atrioventricular (AVSD): Q212, Tetralogy of Fallot: Q213)<br>Defects of pulmonary and tricuspid valves: Q22<br>Defects of mitral and aortic valves: Q23<br>Other cardiac defects: Q240-Q245, Q248-249<br>Defects of great arteries: Q25<br>Defects of the great veins: Q260, Q262-Q269
Respiratory system	Q300, Q321, Q323-Q329, Q330, Q332-Q335, Q337-Q339, Q34
Oral clefts	Cleft palate: Q351, Q353, Q355, Q359<br>Cleft lip with or without cleft palate: Q36-Q37
Digestive system	Q380, Q383-Q389, Q39, Q402-Q409, Q41-Q42, Q431-Q439, Q440-Q443, Q445-Q447, Q45, Q790
Abdominal wall defects	Q792-Q793, Q795
Urinary system	Q60, Q611-Q619, Q620-Q626, Q628-Q629, Q630-Q632, Q634-Q639, Q64, Q794
Genital organs	Q500, Q503, Q504, Q506, Q51, Q520-Q522, Q524, Q526, Q528-Q529, Q540-Q543, Q548-Q549, Q55-Q56
Hypospadias	Q540-Q543, Q548-Q549
Limb	Q650-Q652, Q658-Q659, Q660, Q679, Q681-Q682, Q686-Q689, Q69, Q70-Q74
Other congenital malformations	Q750, Q77, Q782-Q788, Q80-Q81, Q820-Q824, Q826-Q829, Q860, Q890, Q893-Q894
**Indications**	**ICD-10 codes**
GERD	K20, K21
Barrett's esophagus	K227
Ulcer	K25-K28
Gastritis and duodenitis	K29

© 2023 Choi A et al. *JAMA Network Open*.

Dyspepsia	K30
Heartburn	R12
Zollinger-Ellison syndrome	E164
Helicobacter pylori infection	B980
**Maternal conditions**†	**ICD-10 codes**
Anxiety	F40-F41
Diabetes	E10-E14
Epilepsy/seizures	G40-G41
Headache/migraine	G43-G44, R51
Hypertension	I10-I15
Renal disease	E112, E132, E142, I12-I13, N00-N08, N17-N19, N25-N27
Alcohol or drug dependence	F10-F16, F18-F19, Z71.4, Z71.5, Z72.1, Z72.2
Tobacco dependence	F17, Z71.6, Z72.0
Nausea and vomiting	R11, O21
**Obstetric conditions**	**NHIS-NHID procedure codes or ICD 10 codes**
Nulliparous	R3131, R3133, R3141, R3143, R4351, R4353, R4361, R4517, R4519, R4507, R4509, R5001, RA361, RA311, RA312, RA315, RA316, RA431, RA432
Multifetal pregnancy	R3133, R3138, R3143, R3148, R4353, R4358, R4516, R4519, R4520, R5001, R5002, RA312, RA314, RA316, RA318, RA432, RA434
Preterm birth	O42, O601, O603 on mothers' code at delivery date<br>P072, P073 on infants' codes between delivery + delivery 30 days
**Concomitant medications**†	**ATC codes**
Antidepressants	N06A
Antidiabetic drugs	A01A-B
Antihypertensives	C03A-E, C07, C08C-D, C09C-D
Benzodiazepines	N03AE, N05BA, N05CD
Corticosteroids	H02B
Fertility drugs	G03G
Opioid analgesics	N02A
Non-steroidal anti-inflammatory drugs	M01A
Thyroid hormones	H03AA
Antithyroid drugs	H03B
Lipid lowering drug	C10
Stimulants	N06BA
Triptans	N02CC
Fluconazole	J02AC

Abbreviations: ATC=Anatomical Therapeutic Chemical Classification, ICD-10=International Classification of Diseases 10th revision, NHIS-NHID=National Health Insurance Service-National Health Information Database

† Maternal medical conditions and medications used were assessed between the 6 months before pregnancy and the first trimester. The measures of health care use were assessed during the period 6 months prior to pregnancy.

© 2023 Choi A et al. *JAMA Network Open*.

eTable 3. Baseline Characteristics of Pregnant Women Who Delivered Boys for the Analysis of Hypospadias

	Unadjusted				PS-Adjusted		
	No. (%) of Pregnancies		Standardized difference		No. (%) of Pregnancies		Standardized difference
Characteristics	PPI (n= 20,900)	Unexposed (n= 1,371,387)			PPI (n= 20,889)	Unexposed (n= 1,371,386)	
**Age, mean (SD)**	32.4 (4.6)	32.1 (4.2)	0.07		32.4 (4.5)	32.4 (4.5)	-0.01
**Age group**							
19-25	1,489 (7.1)	86,314 (6.3)	0.03		1,489 (7.1)	96,131 (7.0)	0.01
26-30	5,306 (25.4)	368,833 (26.9)	-0.03		5,306 (25.4)	350,140 (25.5)	0.00
31-35	8,846 (42.3)	639,252 (46.6)	-0.09		8,842 (42.3)	584,578 (42.6)	-0.01
36-40	4,472 (21.4)	248,125 (18.1)	0.08		4,466 (21.4)	291,167 (21.2)	0.00
41-44	787 (3.8)	28,863 (2.1)	0.10		786 (3.8)	49,370 (3.6)	0.01
**Medical aid recipients**	289 (1.4)	7,356 (0.5)	0.09		286 (1.4)	17,220 (1.3)	0.01
**Income level**							
1st quartile	4,456 (21.3)	264,408 (19.3)	0.05		4,450 (21.3)	290,161 (21.2)	0.00
2nd quartile	5,068 (24.2)	328,754 (24.0)	0.01		5,067 (24.3)	331,321 (24.2)	0.00
3rd quartile	6,995 (33.5)	480,792 (35.1)	-0.03		6,992 (33.5)	461,557 (33.7)	0.00
4th quartile	4,381 (21.0)	297,433 (21.7)	-0.02		4,380 (21.0)	288,347 (21.0)	0.00
**Region**							
Metropolitan	14,080 (67.4)	959,018 (69.9)	-0.06		14,075 (67.4)	923,096 (67.3)	0.00
Urban	6,819 (32.6)	411,742 (30.0)	0.06		6,813 (32.6)	448,243 (32.7)	0.00
Rural	1 (0.0)	627 (0.0)	-0.03		1 (0.0)	47 (0.0)	0.00
**Nulliparity**	9,813 (47.0)	700,400 (51.1)	-0.08		9,809 (47.0)	646,468 (47.1)	0.00
**Multiple gestation**	559 (2.7)	33,785 (2.5)	0.01		559 (2.7)	36,651 (2.7)	0.00
**Year of delivery**							
2011	984 (4.7)	112,315 (8.2)	-0.14		984 (4.7)	64,984 (4.7)	0.00
2012	2,072 (9.9)	202,790 (14.8)	-0.15		2,070 (9.9)	134,834 (9.8)	0.00
2013	2,074 (9.9)	179,684 (13.1)	-0.10		2,074 (9.9)	135,431 (9.9)	0.00
2014	2,158 (10.3)	178,266 (13.0)	-0.08		2,158 (10.3)	141,321 (10.3)	0.00
2015	2,743 (13.1)	175,676 (12.8)	0.01		2,742 (13.1)	179,033 (13.1)	0.00
2016	2,642 (12.6)	160,911 (11.7)	0.03		2,639 (12.6)	173,742 (12.7)	0.00
2017	2,849 (13.6)	137,226 (10.0)	0.11		2,848 (13.6)	186,943 (13.6)	0.00
2018	2,758 (13.2)	119,770 (8.7)	0.14		2,755 (13.2)	181,742 (13.3)	0.00
2019	2,620 (12.5)	104,749 (7.6)	0.16		2,619 (12.5)	173,357 (12.6)	0.00
**Indications**							
GERD	14,484 (69.3)	74,196 (5.4)	1.76		14,473 (69.3)	951,292 (69.4)	0.00
Barrett's esophagus	8 (0.0)	17 (0.0)	0.03		8 (0.0)	292 (0.0)	0.01
Ulcer	3,802 (18.2)	43,979 (3.2)	0.50		3,792 (18.2)	234,509 (17.1)	0.03
Gastritis and duodenitis	14,328 (68.6)	410,403 (29.9)	0.84		14,318 (68.5)	956,531 (69.7)	-0.03

© 2023 Choi A et al. *JAMA Network Open*.

Dyspepsia	3,583 (17.1)	107,534 (7.8)	0.28		3,574 (17.1)	230,049 (16.8)	0.01
Heartburn	929 (4.4)	31,332 (2.3)	0.12		927 (4.4)	60,691 (4.4)	0.00
Zollinger-Ellison syndrome	0 (0.0)	9 (0.0)	0.00		0 (0.0)	0 (0.0)	0.00
Helicobacter pylori infection	60 (0.3)	170 (0.0)	0.07		55 (0.3)	2,560 (0.2)	0.02
**Medical conditions**							
Anxiety	669 (3.2)	12,459 (0.9)	0.16		666 (3.2)	41,434 (3.0)	0.01
Diabetes	296 (1.4)	9,167 (0.7)	0.07		296 (1.4)	19,086 (1.4)	0.00
Epilepsy	66 (0.3)	2,429 (0.2)	0.03		66 (0.3)	4,143 (0.3)	0.00
Headache/migraine	2,166 (10.4)	66,640 (4.9)	0.21		2,162 (10.3)	141,519 (10.3)	0.00
Hypertension	255 (1.2)	7,700 (0.6)	0.07		255 (1.2)	16,085 (1.2)	0.00
Renal disease	133 (0.6)	3,677 (0.3)	0.06		133 (0.6)	8,687 (0.6)	0.00
Alcohol or drug dependence	45 (0.2)	938 (0.1)	0.04		45 (0.2)	2,966 (0.2)	0.00
Tobacco dependence	2 (0.0)	32 (0.0)	0.01		2 (0.0)	97 (0.0)	0.00
Nausea and Vomiting	4,765 (22.8)	155,530 (11.3)	0.31		4,757 (22.8)	313,028 (22.8)	0.00
**Prescription drug use**							
Antidepressants	1,343 (6.4)	20,417 (1.5)	0.26		1,338 (6.4)	81,789 (6.0)	0.02
Antidiabetic drugs	217 (1.0)	7,590 (0.6)	0.06		217 (1.0)	14,255 (1.0)	0.00
Antihypertensives	941 (4.5)	22,100 (1.6)	0.17		939 (4.5)	59,626 (4.3)	0.01
Benzodiazepines	6,151 (29.4)	111,840 (8.2)	0.57		6,142 (29.4)	391,208 (28.5)	0.02
Corticosteroids	10,847 (51.9)	457,313 (33.3)	0.38		10,837 (51.9)	722,371 (52.7)	-0.02
Fertility drugs	1,191 (5.7)	87,018 (6.3)	-0.03		1,191 (5.7)	80,121 (5.8)	-0.01
Opioid analgesics	12,626 (60.4)	540,438 (39.4)	0.43		12,616 (60.4)	841,383 (61.4)	-0.02
Non-steroidal anti-inflammatory drugs	16,142 (77.2)	799,564 (58.3)	0.41		16,131 (77.2)	1,076,531 (78.5)	-0.03
Thyroid hormones	954 (4.6)	54,016 (3.9)	0.03		954 (4.6)	63,094 (4.6)	0.00
Antithyroid drugs	194 (0.9)	7,727 (0.6)	0.04		194 (0.9)	12,800 (0.9)	0.00
Lipid lowering drug	231 (1.1)	3,470 (0.3)	0.10		230 (1.1)	13,564 (1.0)	0.01
Stimulants	0 (0.0)	30 (0.0)	-0.01		0 (0.0)	2 (0.0)	0.00
Triptans	179 (0.9)	3,330 (0.2)	0.08		176 (0.8)	11,000 (0.8)	0.00
Anti-emetics	6,139 (29.4)	171,154 (12.5)	0.43		6,130 (29.3)	405,121 (29.5)	0.00
Fluconazole	1,348 (6.4)	52,735 (3.8)	0.12		1,346 (6.4)	87,782 (6.4)	0.00
**Obstetric comorbidity index, mean (SD)**	0.7 (1.0)	0.5 (0.8)	0.21		0.7 (1.0)	0.7 (1.0)	0.01
**No. of outpatient visits, mean (SD)**	8.7 (9.4)	5.4 (5.6)	0.43		8.7 (9.3)	8.7 (6.9)	0.01
**No. of emergency room visits, mean (SD)**	0.2 (1.3)	0.1 (0.3)	0.09		0.2 (0.6)	0.2 (0.5)	-0.02
**No. of hospitalizations, mean (SD)**	0.1 (0.5)	0.1 (0.3)	0.13		0.1 (0.5)	0.1 (0.4)	0.00

Abbreviation: PPI=Proton pump inhibitor, SD=Standard deviation, GERD=Gastroesophageal reflux disease

© 2023 Choi A et al. *JAMA Network Open*.

eTable 4. Risk Difference and 95% CIs Comparing the Risk of Congenital Malformations Between Pregnancies Exposed and Not Exposed to Proton Pump Inhibitors (PPIs)

	PPI (n=40,540)			Unexposed (n=2,655,676)			Unadjusted Risk Difference per 10,000* (95% CI)	Adjusted Risk Difference per 10,000* (95% CI)
	Events		Risk per 10,000*	Events		Risk per 10,000*		
Major congenital malformations	1,608		396.6	85,900		323.5	73.19 (54.07-92.30)	26.62 (6.04-47.21)
Congenital heart defects	851		209.9	42,154		158.7	51.18 (37.15-65.22)	17.73 (2.60-32.86)
Cleft palate	38		9.4	2,281		8.6	0.78 (-2.22-3.78)	0.14 (-3.08-3.36)
Hydrocephalus	13		3.2	784		3.0	0.25 (-1.50-2.01)	-0.20 (-1.96-1.57)
Hypospadias†	25		12.0	1,799		13.1	-1.16 (-5.88-3.57)	-3.56 (-8.82-1.70)

Abbreviations: PPI=Proton Pump Inhibitor, CI=Confidence Interval

*per 10,000 infants

†Estimated among 20,900 pregnancies exposed to PPIs and 1,371,387 unexposed pregnancies that gave birth to boys.

© 2023 Choi A et al. *JAMA Network Open*.

eTable 5. Association Between Proton Pump Inhibitor (PPI) Exposure in Pregnancy and the Risk of Subtype of Major Congenital Malformations

	PPI (n=40,540)	Unexposed (n=2,655,676)	Unadjusted Risk Difference per 10,000* (95% CI)	Adjusted Risk Difference per 10,000* (95% CI)	Unadjusted Relative Risk (95% CI)	Adjusted Relative Risk (95% CI)
**Subgroups of congenital malformations**						
Nervous system	103	4,685	7.77 (2.84-12.69)	5.04 (-0.22-10.31)	1.44 (1.19-1.75)	1.24 (0.99-1.54)
Eye	34	2,058	0.64 (-2.20-3.48)	-0.37 (-3.47-2.72)	1.08 (0.77-1.52)	0.96 (0.66-1.38)
Ear, face, and neck	6	431	-0.14 (-1.34-1.05)	-0.68 (-2.05-0.68)	0.91 (0.41-2.04)	0.68 (0.29-1.61)
Congenital heart defects	851	42,154	51.18 (37.15-65.22)	17.73 (2.60-32.86)	1.32 (1.24-1.41)	1.09 (1.01-1.17)
Respiratory system	25	866	2.91 (0.48-5.33)	2.68 (0.14-5.23)	1.89 (1.27-2.81)	1.77 (1.12-2.79)
Oral clefts	49	3,220	-0.04 (-3.45-3.37)	-0.75 (-4.45-2.96)	1.00 (0.75-1.32)	0.94 (0.70-1.28)
Digestive system	90	4,602	4.87 (0.26-9.48)	4.48 (-0.42-9.38)	1.28 (1.04-1.58)	1.25 (0.98-1.57)
Abdominal wall defects	6	230	0.61 (-0.58-1.80)	0.94 (-0.26-2.14)	1.71 (0.76-3.84)	2.74 (1.14-6.57)
Urinary system	179	11,188	2.03 (-4.48-8.53)	-1.59 (-8.62-5.45)	1.05 (0.90-1.21)	0.97 (0.82-1.13)
Genital organs	41	2,548	0.52 (-2.60-3.64)	-0.37 (-3.74-3.00)	1.05 (0.77-1.43)	0.96 (0.69-1.34)
Limb	238	13,463	8.01 (0.53-15.50)	4.07 (-3.96-12.09)	1.16 (1.02-1.31)	1.07 (0.94-1.23)
Others	56	3,527	0.53 (-3.11-4.17)	-0.98 (-4.95-2.98)	1.04 (0.80-1.35)	0.93 (0.70-1.24)

Abbreviations: PPI=Proton Pump Inhibitor, CI=Confidence Interval
*per 10,000 infants

© 2023 Choi A et al. *JAMA Network Open*.

eTable 6. Relative Risks and 95% CIs Comparing the Risk of Subtypes of Congenital Heart Defects Between Pregnancies Exposed and Not Exposed to Proton Pump Inhibitors (PPIs)

	PPI (n=40,540)				Unexposed (n=2,655,676)			Unadjusted Relative Risk (95% CI)		Adjusted Relative Risk (95% CI)
	Events		Risk per 10,000*		Events		Risk per 10,000*			
**Subgroups of congenital heart defects**										
Defects of cardiac chambers and their connections	21		5.2		1,230		4.6	1.12 (0.73-1.72)		0.98 (0.61-1.56)
Septal defects	679		167.5		33,227		125.1	1.34 (1.24-1.44)		1.09 (1.01-1.19)
Defects of pulmonary and tricuspid valves	36		8.9		1,639		6.2	1.44 (1.03-2.00)		1.25 (0.87-1.79)
Defects of mitral and aortic valves	10		2.5		481		1.8	1.36 (0.73-2.55)		0.95 (0.48-1.86)
Other cardiac defects	11		2.7		993		3.7	0.73 (0.40-1.31)		0.69 (0.37-1.29)
Defects of great arteries	128		31.6		6,513		24.5	1.29 (1.08-1.53)		1.12 (0.92-1.35)
Defects of the great veins	7		1.7		382		1.4	1.29 (0.57-2.93)		1.20 (0.57-2.53)

Abbreviations: PPI=Proton Pump Inhibitor, CI=Confidence Interval
*per 10,000 infants

© 2023 Choi A et al. *JAMA Network Open*.

eTable 7. Risk Differences and 95% CIs Comparing the Risk of Subtypes of Congenital Heart Defects Between Pregnancies Exposed and Not Exposed to Proton Pump Inhibitors (PPIs)

	PPI (n=40,540)		Unexposed (n=2,655,676)		Unadjusted Risk Difference per 10,000* (95% CI)	Adjusted Risk Difference per 10,000* (95% CI)
	Events	Risk per 10,000*	Events	Risk per 10,000*		
**Subgroups of congenital heart defects**						
Defects of cardiac chambers and their connections	21	5.2	1,230	4.6	0.55 (-1.68-2.78)	-0.12 (-2.56-2.33)
Septal defects	679	167.5	33,227	125.1	42.37 (29.81-54.94)	15.11 (1.57-28.65)
Defects of pulmonary and tricuspid valves	36	8.9	1,639	6.2	2.71 (-0.21-5.62)	1.75 (-1.37-4.87)
Defects of mitral and aortic valves	10	2.5	481	1.8	0.66 (-0.88-2.19)	-0.13 (-1.82-1.55)
Other cardiac defects	11	2.7	993	3.7	-1.03 (-2.65-0.59)	-1.24 (-3.06-0.57)
Defects of great arteries	128	31.6	6,513	24.5	7.05 (1.56-12.54)	3.28 (-2.63-9.19)
Defects of the great veins	7	1.7	382	1.4	0.39 (-0.97-1.75)	0.29 (-1.00-1.58)

Abbreviations: PPI=Proton Pump Inhibitor, CI=Confidence Interval
*per 10,000 infants

© 2023 Choi A et al. *JAMA Network Open*.

eTable 8. Association Between Proton Pump Inhibitor (PPI) Exposure in Pregnancy and the Risk of Congenital Malformations Among Sibling Populations

	Unadjusted Relative Risk (95% CI)	Adjusted Relative Risk (95% CI)
Major congenital malformations	1.21 (1.12-1.30)	1.06 (0.98-1.16)
Congenital heart defects	1.32 (1.19-1.47)	1.08 (0.97-1.22)
Cleft palate	0.85 (0.48-1.49)	0.89 (0.49-1.62)
Hydrocephalus	1.54 (0.73-.3.27)	1.27 (0.60-2.70)

Abbreviations: PPI, Proton pump inhibitor; CI, confidence interval

© 2023 Choi A et al. *JAMA Network Open*.

eTable 9. Odds Ratios of Proton Pump Inhibitor (PPI) Exposure During the Second Pregnancy According to Congenital Malformation Status of the First Sibling

Congenital malformation status in the first sibling	OR (95% CI)[a]
Major congenital malformations	
No	Ref
Yes	1.04 (0.90-1.21)
Congenital heart defects	
No	Ref
Yes	1.09 (0.92-1.30)
Cleft palate	
No	Ref
Yes	1.06 (0.73-1.69)
Hydrocephalus	
No	Ref
Yes	0.95 (0.46-1.90)

Abbreviations: PPI, Proton pump inhibitor; OR, Odds ratio; CI, confidence interval
[a]adjusted for PPI exposure during the first pregnancy.

© 2023 Choi A et al. *JAMA Network Open*.

eFigure 1. Study Flowchart

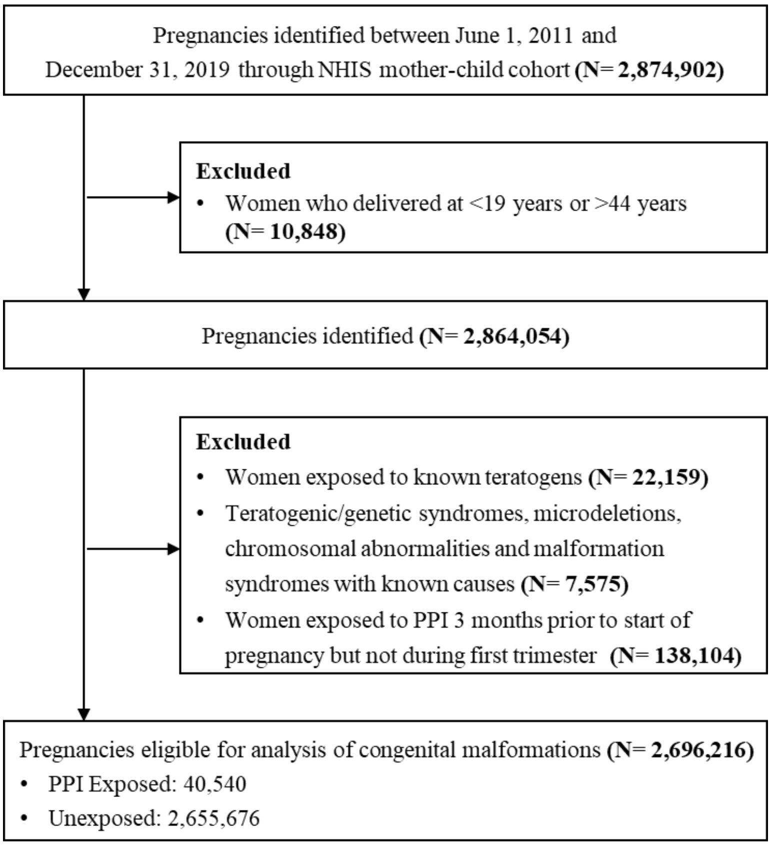

Abbreviations: NHIS= National Health Insurance Service, PPI=Proton Pump Inhibitor

© 2023 Choi A et al. *JAMA Network Open*.

eFigure 2. Frequency of Pregnancies Exposed to Proton Pump Inhibitors (PPIs) During the First Trimester

Specific PPIs	Pregnancies exposed to PPIs during the first trimester, N(%)									
	2011	2012	2013	2014	2015	2016	2017	2018	2019	**Total**
**Total no. of pregnancies**	219,216	396,569	353,024	350,334	346,420	317,169	270,476	237,517	207,741	2,696,216
**Pregnancies exposed to PPIs**	2,000 (0.91)	4,100 (1.03)	4,206 (1.19)	4,465 (1.27)	5,513 (1.59)	5,491 (1.73)	5,898 (2.18)	5,694 (2.40)	5,423 (2.61)	40,540 (1.50)
Rabeprazole	688	1,385	1,568	1,571	1,800	1,565	1,664	1,679	1,494	13,414 (0.50)
Esomeprazole	261	631	721	1,006	1,534	1,877	2,122	2,119	2,245	12,516 (0.46)
Lansoprazole	375	781	741	699	866	847	780	698	617	6,404 (0.24)
Pantoprazole	357	682	616	637	624	595	723	615	544	5,393 (0.20)
Omeprazole	306	599	495	409	387	295	270	250	213	3,224 (0.12)
Dexlansoprazole	0	0	1	82	177	204	214	196	185	1,059 (0.04)
Ilaprazole	13	22	64	61	125	108	125	137	125	780 (0.03)

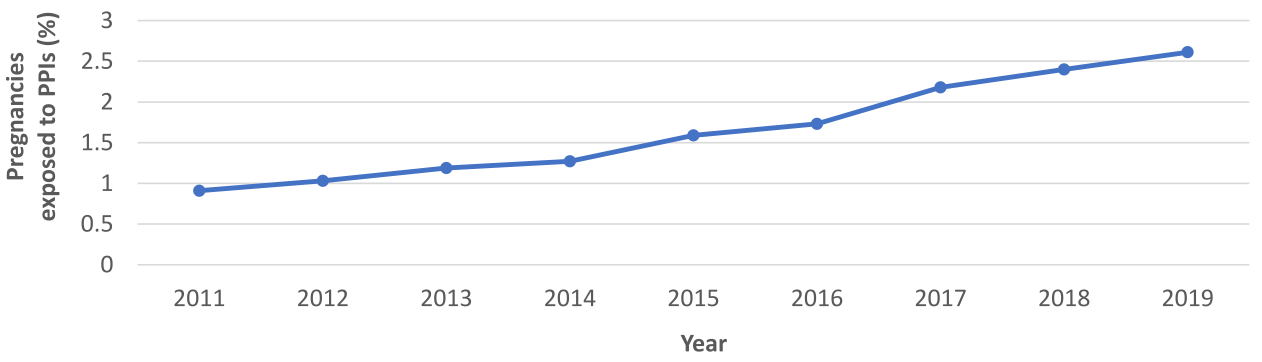

Abbreviation: PPI=Proton Pump Inhibitor

© 2023 Choi A et al. *JAMA Network Open*.

eAppendix 1. Outcome Definition

To increase the specificity, we defined the presence of congenital malformations based on the following algorithms.

Major congenital malformations:

- Presence of any of the malformations stated below

12 System-specific malformations (e.g. congenital heart defects):

- ≥2 visits with any of the codes for organ-specific malformation as a primary diagnosis within one year after delivery (regardless of inpatient or outpatient visits)
- ≥1 visit with a code for organ-specific malformation as a primary diagnosis and malformation-specific surgery/procedure code within one year after delivery
- ≥1 visit with a code for organ-specific malformation as a primary diagnosis and death within one year after delivery

Subgroup of system-specific malformations (e.g. cleft palate, hydrocephalus, hypospadias):

- ≥2 visits with any of the codes for the subgroup of organ-specific malformation as a primary diagnosis within one year after delivery (regardless of inpatient or outpatient visits)
- ≥1 visit with a code for the subgroup of organ-specific malformation as a primary diagnosis and malformation-specific surgery/procedure code within one year after delivery
- ≥1 visit with a code for the subgroup of organ-specific malformation as a primary diagnosis and death within one year after delivery

To further evaluate the reliability of our outcome definition, we tested whether we could reproduce the known associations between pre-existing diabetes and the congenital malformations in our data.[1] Pre-existing diabetes was defined as having 2 or more diagnostic codes between 180 days prior to the start of pregnancy and the first trimester. As a result, we observed significant associations for both major congenital malformations (RR 2.00, 95% CI 1.89-2.12) and congenital heart defects (2.60, 2.42-2.79). We also conducted additional analyses that restricted the outcome definition to inpatient diagnoses only and the estimates were consistent with our main result (major congenital malformation: adjusted RR 1.07, 95% CI 0.99-1.15; congenital heart defects: 1.03, 0.93-1.14).

Reference

1. Bateman, Brian T., et al. "Statins and congenital malformations: cohort study." *bmj* 350 (2015).

© 2023 Choi A et al. *JAMA Network Open*.

eAppendix 2. Additional Details on the Sibling Analysis

To account for potential confounding from family-related factors, we additionally performed sibling-controlled analyses. In the sibling analysis, shared familial/genetic factors within the family could be adjusted by comparing the risk of outcome among the infants born to the same mother. In our study, we included women with at least two pregnancy episodes during the study period. Among those pregnancies, only the siblings who were discordant for both exposure and outcome contributed to the estimates. Because the estimation depends on the discordant sibling pairs, multifetal pregnancies did not contribute to the estimate as they share the same exposure status. Using the logistic regression models stratified on the mother's unique identifier, odds ratio (OR) with 95% confidence intervals (CIs) were estimated adjusting for all covariates considered in the main analysis. The results are presented in Figure 3 and because there were zero cases observed among the exposed male siblings, the risk of hypospadias could not be estimated.

Additional analyses were done to test the assumptions of the sibling analysis:

First, to confirm whether the results from sibling populations are generalizable to the full population, we reran the analysis among the sibling populations while not using the stratified term. Overall, we found comparable results between the sibling populations and the full population in our main analysis (**eTable 8**).

Second, the sibling analysis assumes no carryover effect, that is the outcome of the first sibling should not influence the exposure status of the second sibling[1]. To test the presence of carryover effect, we analyzed the association between the congenital malformation status of the first sibling and PPI exposure during the second pregnancy. The OR of PPI exposure during the second pregnancy was estimated according to the malformation status of the first sibling, while controlling for PPI exposure during the first pregnancy. As a result, we obtained odds ratio approximate to 1 for all our outcomes, indicating that the carryover effect is most likely absent (**eTable 9**).

Reference

1. Sjölander, Arvid, et al. "Carryover effects in sibling comparison designs." Epidemiology 27.6 (2016): 852-858.

eAppendix 3. Potential Consequences of Including Only Live Births

Commented [AC1]: We wonder whether this order would be better. (since eTable 10 belongs to eAppendix 3).

Our study cohort included pregnancies that resulted in live births and did not include pregnancies that ended in stillbirth or abortions. This restriction may introduce selection bias if the probability of live birth differs between PPI-exposed and unexposed pregnancies. For instance, if the probability of live birth is lower in PPI-exposed pregnancies than in unexposed pregnancies owing to a higher rate of pregnancy terminations due to severe malformations, then the risk estimates may be biased toward the null. Therefore, we quantified the potential effects of missing non-live births.

Considering the different probability of live births between PPI-exposed and unexposed pregnancies, the corrected relative risks were estimated as below. This method has been widely used in previous studies to quantify the potential effect of restriction to live births [1,2]:

Corrected relative risk (RR)=Observed RR*(S_{10}*S_{01}/S_{11}*S_{00})

S_{10} refers to the probability of live births in PPI-unexposed pregnancies with malformation.
S_{01} refers to the probability of live births in PPI-exposed pregnancies without malformation.
S_{11} refers to the probability of live births in PPI-exposed pregnancies with malformation.
S_{00} refers to the probability of live births in PPI-unexposed pregnancies without malformation.

Based on estimates from the literature, the live birth probability among PPI-unexposed pregnancies without malformation (S_{00}) was defined as 80%.[3] We then assumed the probability of live birth among unexposed pregnancies with malformations (S_{10}) as a range of 55% to 80%, based on a previous study.[4] Lastly, we evaluated the potential effect of lower frequency of live births, ranging from 10% to 20%, in PPI exposed pregnancies (**eTable 10**).

eTable 10. Probability of Live Births

**Pregnancies with malformations**		**Pregnancies without malformations**	
**Unexposed (S_{10})**	**PPI exposed (S_{11})**	**Unexposed (S_{00})**	**PPI exposed (S_{01})**
55–80%	S_{10}-20%	80%	S_{00}-20%
	S_{10}-10%		S_{00}-10%
	S_{10}		S_{00}

Abbreviation: PPI=Proton Pump Inhibitor

References

1. Huybrechts KF, Palmsten K, Avorn J, et al. Antidepressant use in pregnancy and the risk of cardiac defects. New England Journal of Medicine 2014;370(25):2397-407.
2. Patorno E, Huybrechts KF, Bateman BT, et al. Lithium use in pregnancy and the risk of cardiac malformations. New England Journal of Medicine 2017;376(23):2245-54.
3. Lee S-Y. 2018 National Survey on Fertility, Family Health and Welfare: Korea Institute for Health and Social Affairs, 2019.
4. Svensson E, Ehrenstein V, Nørgaard M, et al. Brief Report: Estimating the Proportion of All Observed Birth Defects Occurring in Pregnancies Terminated by a Second-trimester Abortion. Epidemiology 2014:866-71.

© 2023 Choi A et al. *JAMA Network Open*.

eFigures 3 and 4 show the corrected RR for major congenital malformations and congenital heart defects, respectively. Starting from the estimates in our main analysis (RR = 1.07 for major congenital malformations and 1.09 for congenital heart defects), the risk remained below 1.3, under the most extreme assumption (assuming the selection probability of PPI-exposed pregnancies with malformation as 35%).

© 2023 Choi A et al. *JAMA Network Open*.

eFigure 3. Corrected Relative Risk for the Association Between Proton Pump Inhibitor (PPI) Exposure During the First Trimester and Major Congenital Malformations

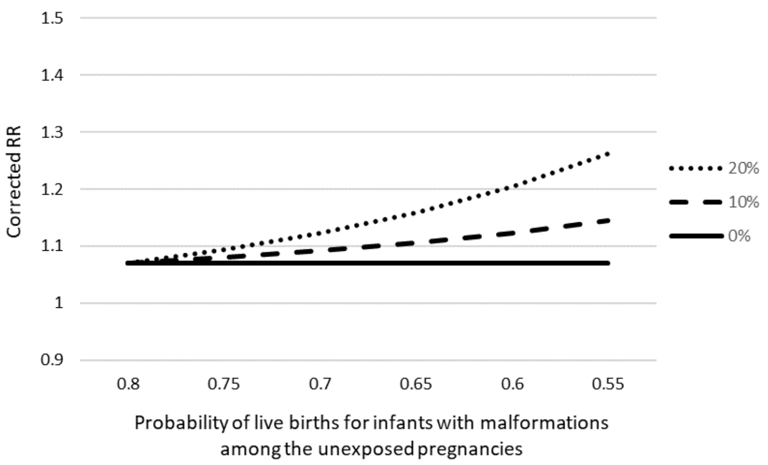

© 2023 Choi A et al. *JAMA Network Open*.

eFigure 4. Corrected Relative Risk for the Association Between Proton Pump Inhibitor (PPI) Exposure During the First Trimester and Congenital Heart Defects

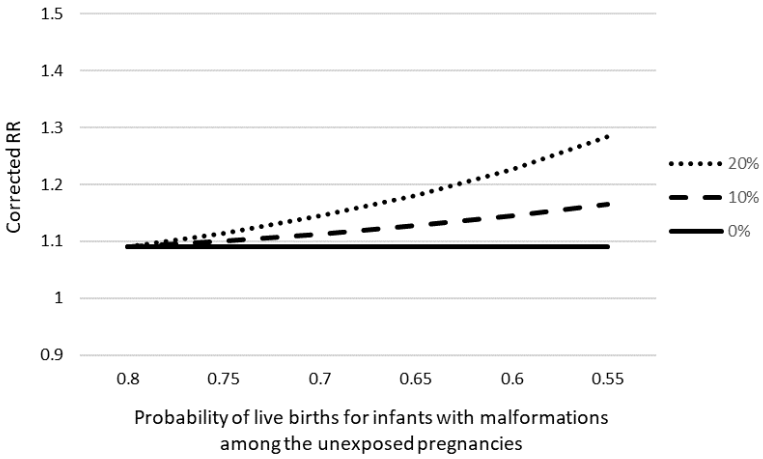

Abbreviations: PPI=Proton Pump Inhibitor, RR= RR=Relative Risk

© 2023 Choi A et al. *JAMA Network Open*.
