# Supplementary material for: Association Between Proton Pump Inhibitor Use During Early Pregnancy and Risk of Congenital Malformations
Source: JAMA Netw Open. 2023 Jan 10;6(1):e2250366. doi: 10.1001/jamanetworkopen.2022.50366 (PMC9856708; doi:10.1001/jamanetworkopen.2022.50366)
Supplement: Supplement 2. — Data Sharing Statement [file jamanetwopen-e2250366-s002.pdf]

Data Sharing Statement

Choi. Association Between Proton Pump Inhibitor Use During Early Pregnancy and Risk of Congenital Malformations. *JAMA Netw Open*. Published January 10, 2023. doi:10.1001/jamanetworkopen.2022.50366

Data
Data available: No
